# Supplementary material for: Low-defect-density WS2 by hydroxide vapor phase deposition
Source: Nat Commun. 2022 Jul 18;13:4149. doi: 10.1038/s41467-022-31886-0 (PMC9293887; doi:10.1038/s41467-022-31886-0)
Supplement: Supplementary file 1 — Supplementary Information [file 41467_2022_31886_MOESM1_ESM.pdf]

Low-defect-density WS_2 by hydroxide vapor phase deposition

Yi Wan[1,2], En Li[3], Zhihao Yu[4,5], Jing-Kai Huang[6], Ming-Yang Li[4], Ang-Sheng Chou[4], Yi-Te Lee[7], Chien-Ju Lee[7], Hung-Chang Hsu[8], Qin Zhan[9], Areej Aljarb[10], Jui-Han Fu[1,11], Shao-Pin Chiu[7], Xinran Wang[5], Juhn-Jong Lin[7], Ya-Ping Chiu[8], Wen-Hao Chang[7,12], Han Wang[4], Yumeng Shi[13], Nian Lin[3], Yingchun Cheng[9]*, Vincent Tung[1,11]*, Lain-Jong Li[2*]

[1]Physical Sciences and Engineering Division, King Abdullah University of Science and Technology (KAUST), Thuwal, Kingdom of Saudi Arabia

[2]Department of Mechanical Engineering, The University of Hong Kong, Pokfulam Road, Hong Kong

[3]Department of Physics, The Hong Kong University of Science and Technology, Hong Kong

[4]Corporate Research, Taiwan Semiconductor Manufacturing Company (TSMC), 168 Park Ave. 2, Hsinchu Science Park, Hsinchu 30075, Taiwan.

[5]National Laboratory of Solid State Microstructures, School of Electronic Science and Engineering and Collaborative Innovation Center of Advanced Microstructures, Nanjing University, Nanjing 210093, China

[6]School of Materials Science and Engineering, University of New South Wales, Sydney, New South Wales, Australia

[7]Department of Electrophysics, National Yang Ming Chiao Tung University, Hsinchu 30010, Taiwan.

[8]Department of Physics, National Taiwan University, Taipei 10617, Taiwan

[9]Key Laboratory of Flexible Electronics & Institute of Advanced Materials, Nanjing Tech University, Nanjing, 211816, China

[10]Department of Physics, King Abdulaziz University (KAAU), Jeddah 23955-6900, Saudi Arabia

[11]Department of Chemical System and Engineering, The University of Tokyo, Tokyo, 113-8654, Japan

[12]Research Center for Applied Sciences, Academia Sinica, Taipei 11529, Taiwan

[13]International Collaborative Laboratory of 2D Materials for Optoelectronic Science & Technology of Ministry of Education, Engineering Technology Research Center for 2D Material Information Function Devices and Systems of Guangdong Province, College of Optoelectronic Engineering, Shenzhen University, Shenzhen, 518060, China

*To whom correspondence should be addressed: lanceli1@hku.hk or vincent.tung@kaust.edu.sa or iamyccheng@njtech.edu.cn

Cotents

I. Supplementary Notes

Supplementary Note 1. OHVPD growth mechanism
Supplementary Note 2. PL and Raman mapping results
Supplementary Note 3. Discussion of OHVPD growth condition

II. Supplementary Figures

Supplementary Figure 1. Reaction of W metals with H_2O and/or H_2 by XRD
Supplementary Figure 2. Hydrogen effect investigation in the OHVPD process
Supplementary Figure 3. Kinetic simulation of sulfurization process
Supplementary Figure 4. PL and Raman mapping of OHVPD-WS_2 monolayer
Supplementary Figure 5. Photoluminescence comparison between CVD- and OHVPD-WS_2 monolayers
Supplementary Figure 6. OHVPD-MoS_2
Supplementary Figure 7. STM/STS for sulfur vacancy
Supplementary Figure 8. STM/STS for O_S defect
Supplementary Figure 9. STM/STS for negatively charged defect (NCD)
Supplementary Figure 10. Simulation of O_S defect formation
Supplementary Figure 11. Electrical performance of CVD-WS_2 monolayers
Supplementary Figure 12. Theoretical fitting of μ-T characteristic for OHVPD- and CVD-WS_2 devices
Supplementary Figure 13. Summary of reported N_{tr} and N_{CI} for MoS_2 and WS_2 monolayers with different fabrication technologies
Supplementary Figure 14. Typical dual-sweep transfer curve characteristics of OHVPD-WS_2 monolayer device
Supplementary Figure 15. Benchmarking field-effect mobility for WS_2 monolayers based on two-probe measurements
Supplementary Figure 16. The output characteristics of the short-gate-length (100 nm) FET based on OHVPD-WS_2.
Supplementary Figure 17. Temperature and PL distributions
Supplementary Figure 18. Water vapor supply influence

III. Supplementary Tables

Supplementary Table 1. Benchmarking FET performance of monolayer TMDCs

IV. Supplementary References

I. Supplementary Notes

Supplementary Note 1: OHVPD growth mechanism.

To reveal the WS_2 growth mechanism with OHVPD, the W foils were heated in different gas environments and characterized by X-ray Diffraction (XRD). Firstly, the W metal foil was annealed at 1050°C, where the H_2O was brought to the chamber with Ar purge (180 sccm) at atmospheric pressure. After cooling to room temperature, the XRD spectrum was collected (**Supplementary Figure 1a),** and the XRD result displays both W peaks and complex tungsten oxides (WO_x) peaks which include $W_{18}O_{49}$ and tungstite ($WO_2(OH)_2$). The oxidation reaction steps of W metal with moisture gas for the WS_2 growth can be written as following[1]:

$$\mathrm{W}(s) + \mathrm{H_2O(g)} \rightleftharpoons 2\mathrm{H_2(g)} + \mathrm{WO_2}(s) \tag{1}$$

$$\mathrm{WO_2}(s) + \mathrm{H_2O(g)} \rightleftharpoons \mathrm{WO_3}(s) + \mathrm{H_2(g)} \tag{2}$$

$$\mathrm{WO_2}(s) + 2\mathrm{H_2O(g)} \longrightarrow \mathrm{WO_2(OH)_2(g)} + \mathrm{H_2(g)} \tag{3}$$

$$\mathrm{WO_3}(s) + \mathrm{H_2O(g)} \longrightarrow \mathrm{WO_2(OH)_2(g)} \tag{4}$$

A separate and comparative experiment was performed to understand the role of H_2 gas in the reaction, where a W foil was annealed at the same temperature with an extra H_2 gas (10%), and the XRD result was in **Supplementary Figure 1b**. We note that the XRD spectrum only exhibited pure W peaks, which proved that the H_2 gas has an inhibition effect for W-H_2O reaction[2]. However, it is noteworthy that when the percentage of H_2 is low (e.g., 1% as shown in **Supplementary Figure 2**), the W metal turned black and was covered with WO_2 after the annealing. Because the reaction (1) and (2) are reversible,

reducing hydrogen (or equivalently adding sulfur precursors) shall drive the reactions toward the left, leading to the formation of tungsten-oxides like WO_2, WO_3, $W_{18}O_{49}$, or $WO_2(OH)_2$. Since $WO_2(OH)_2$ is more volatile[3] and $W_{18}O_{49}$ is the most refractory tungsten oxide[3], the dominated species observed after the entire reaction should be $W_{18}O_{49}$, as shown in **Supplementary Figure 1a**.

Supplementary Note 2: PL and Raman mapping results.

To present the sample homogeneity, PL and Raman mapping of 2×2 mm^2 WS_2 film were performed as shown in **Supplementary Figure 4**. By using a 488 nm excitation laser, the E_{2g} and A_{1g} Raman modes of WS_2 are easier to clarify during the mapping process. As shown in **Supplementary Figure 4b and c**, the average intensity ratio of $I_{E_{2g}}/I_{A_{1g}}$ is 0.7, and the average frequencies difference between E_{2g} and A_{1g} is 61.5 cm^{-1} which corresponds to WS_2 monolayer thickness.[4] PL intensity and peak position mapping images (**Supplementary Figure 4d and e**) show that the average emission peak position of the film is around 620 nm (2 eV) with identical PL intensity. It is hard to avoid seeds and multilayer growth in the large-scale deposition process (**Supplementary Figure 4f**), so a small quantity of brighter or darker dots can be found in the mapping image. Similar to other reports[5], a higher density of multilayers and seeds would be found at the center of the film compared to the edge. Further study is needed to decrease these seeds and multilayers.

Supplementary Note 3: Discussion of OHVPD growth condition.

1. Temperature gradient of the substrate

Supplementary Figure 17a shows the temperature gradient of our single-heating zone furnace when the heating temperature is set at 1050℃. The position marked as 0 cm corresponds to the center of the furnace. The substrates were distanced from the center by 9 to 14 cm (the temperature range is around 950-800℃). **Supplementary Figure 17b** presents the typical PL peaks of OHVPD-WS_2 collected from different temperature regions. It is evident that the PL peaks of WS_2 film had a redshift when the substrate was put in a higher temperature region (region A). Given that very limited defective and doping variations are concluded from the low-temperature PL and STM results, the possible explanation is that the OHVPD-WS_2 film grown in a higher temperature zone contains higher tensile strain, which results in a PL peak redshift.[6] On the other hand, as concluded in **Supplementary Note 2**, the temperature gradient does not lead to noticeable variation in morphology and thickness of samples because the OHVPE method provides more volatile W-OH reactants that are not hampered by large temperature drops.

2. Water concentration effect

We adopted a bubbler set up to introduce the water vapor into the growth chamber as shown in **Supplementary Figure 18a**. During the experiment, we fixed the carrier gas flow of Ar and adjusted the water temperature using a thermal bath to change the water vapor concentration. By using Antoine equation[7], the partial pressure of water (p_{H_2O}) could be estimated. **Supplementary Figure 18b-d** show the OHVPD-WS_2 growth results with various p_{H_2O}. High p_{H_2O} (92.59 torr, 50℃) results in over oxidization during the reaction, where excess oxides can be found on as-grown samples (**Supplementary Figure 18b**).

In contrast, low p_{H_2O} (17.54 torr, 20℃) lead to a low deposition with small grains due to scarcity of metal supply (**Supplementary Figure 18c**). The optimized water temperature for WS_2 deposition is 35℃ which provides p_{H_2O} = 42.20 torr (**Supplementary Figure 18d**).

II. Supplementary Figures

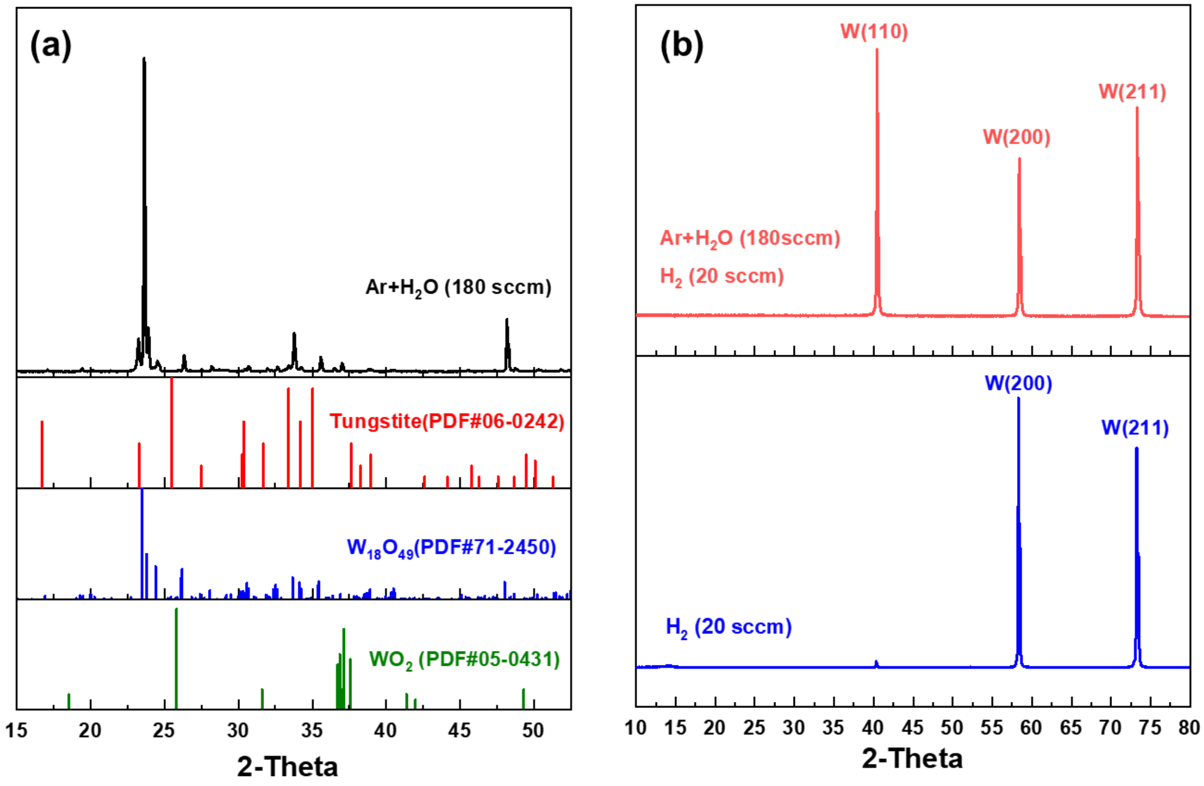

Supplementary Figure 1. Reaction of W metals with H_2O and/or H_2 by XRD. (a) XRD spectrum of the W metals annealed in the environment with H_2O. (b) XRD spectrum of the W metals annealed in the environment with H_2O/H_2 and only H_2.

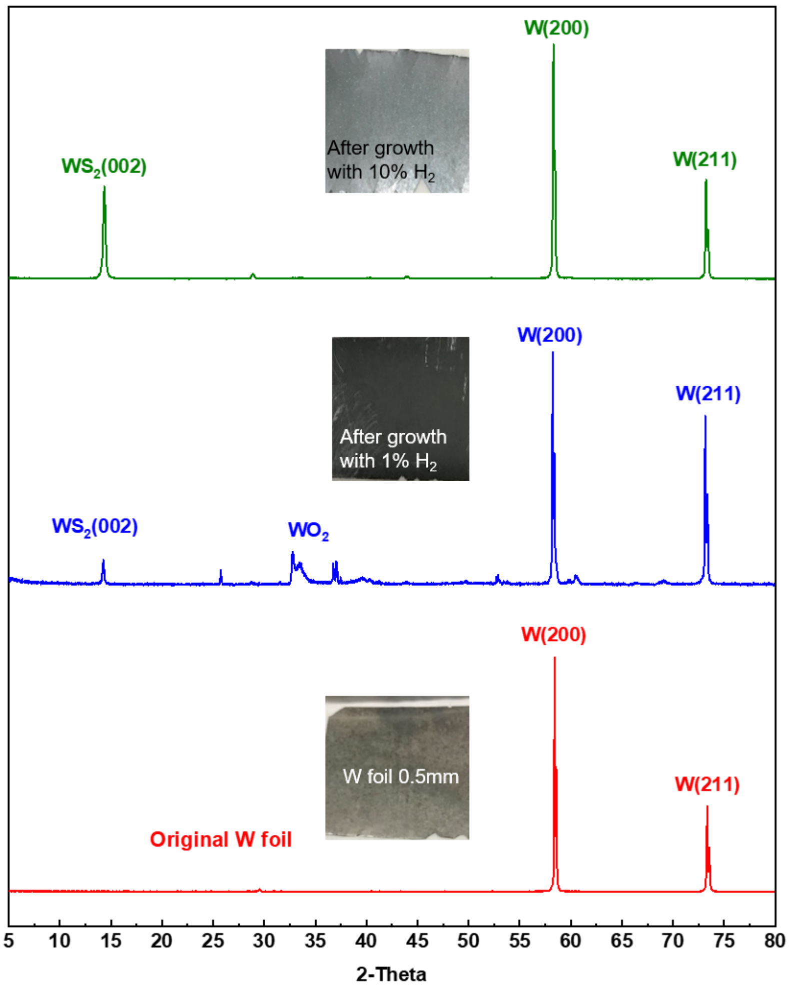

Supplementary Figure 2. Hydrogen effect investigation in the OHVPD process. XRD spectrums were taken from the W metal after the reaction under different H_2 gas concentrations.

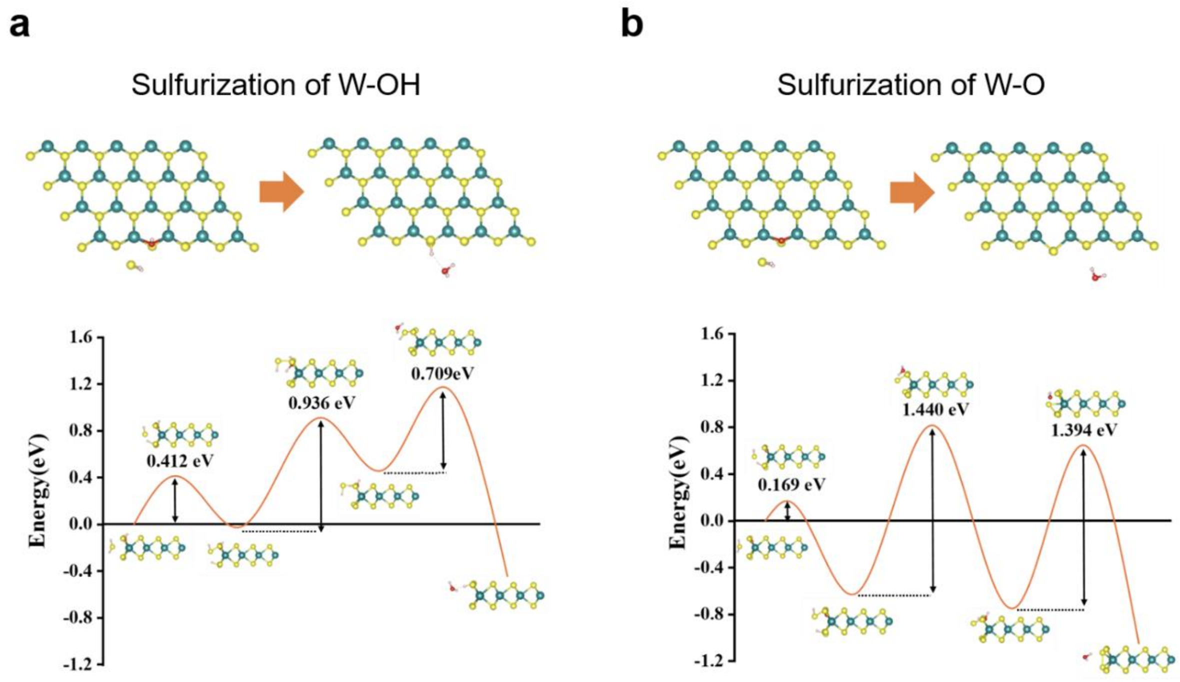

Supplementary Figure 3. Kinetic simulation of sulfurization process. Scheme shows the simulated kinetic energy barriers of sulfurization process for W-OH (a) and W-O (b) in WS_2 crystal.

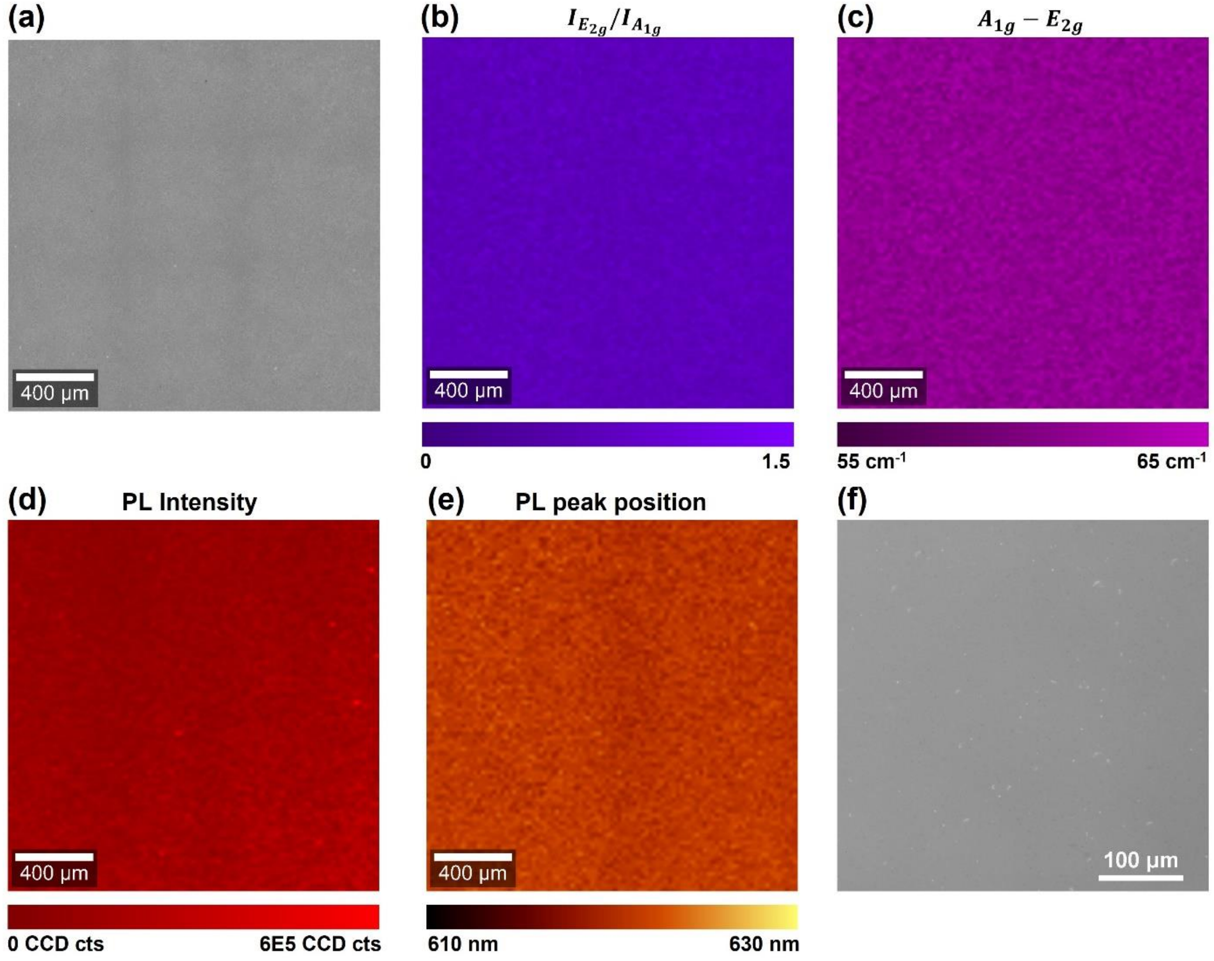

Supplementary Figure 4. PL and Raman mapping of OHVPD-WS_2 monolayer. Optical image (a), Raman mapping results (b, c), and PL mapping results (d, e) of 2 x 2 mm^2 OHVPD-WS_2 film. (f) Optical image shows small quantities of WS_2 seeds and multilayers**.**

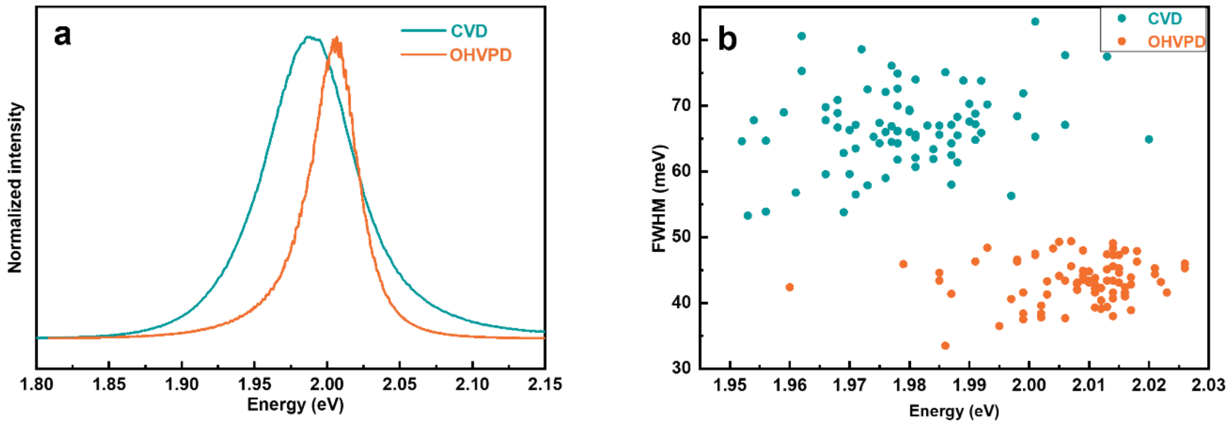

Supplementary Figure 5. Photoluminescence comparison between CVD- and OHVPD-WS_2 monolayers. (a), Typical PL spectra of OHVPD- and CVD- WS_2 monolayers taken at room temperature. (b), Statistic results of PL peak energy and FWHM for OHVPD- and CVD-WS_2 monolayers. For each case, we display a statistical summary of PL spectra for at least 50 single crystal flakes from different growth batches.

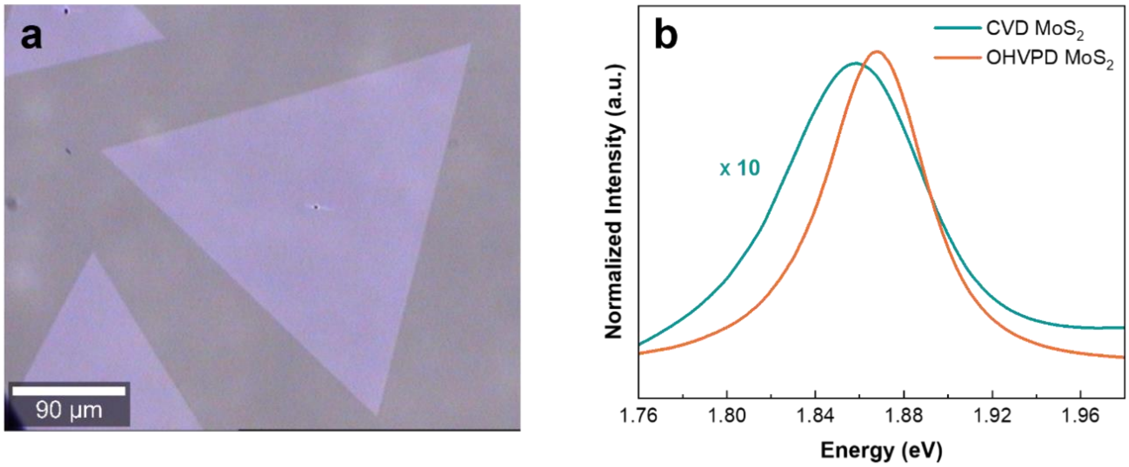

Supplementary Figure 6. OHVPD-MoS_2. (a) Optical image of OHVPD-MoS_2 monolayers. (b) Typical room temperature PL spectra of OHVPD- and CVD-MoS_2.

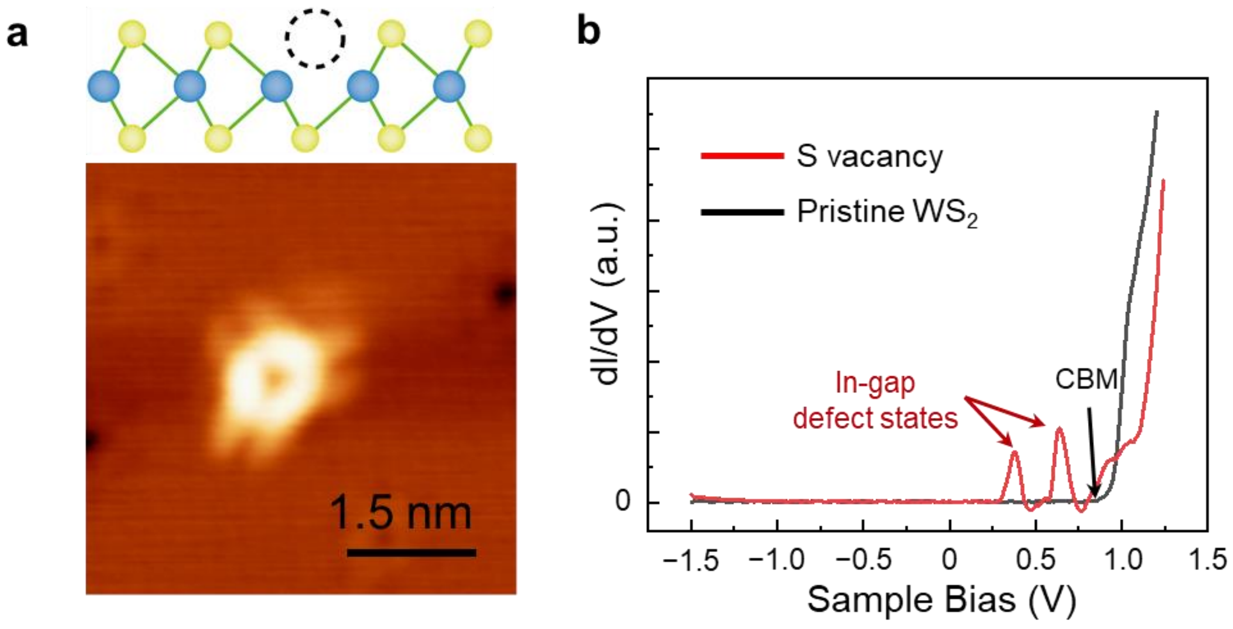

Supplementary Figure 7. STM/STS for sulfur vacancy. (a) Top: a schematic side view of a sulfur vacancy structure. Bottom: an STM topographic image (V=1.1V, I=40pA) of sulfur vacancy point defect in CVD-WS_2 samples. (b) dI/dV spectrums acquired at the center of sulfur vacancy (red curve) and pristine WS_2 region (black curve), showing two narrow unoccupied in-gap defect states below the CBM at the sulfur vacancy.

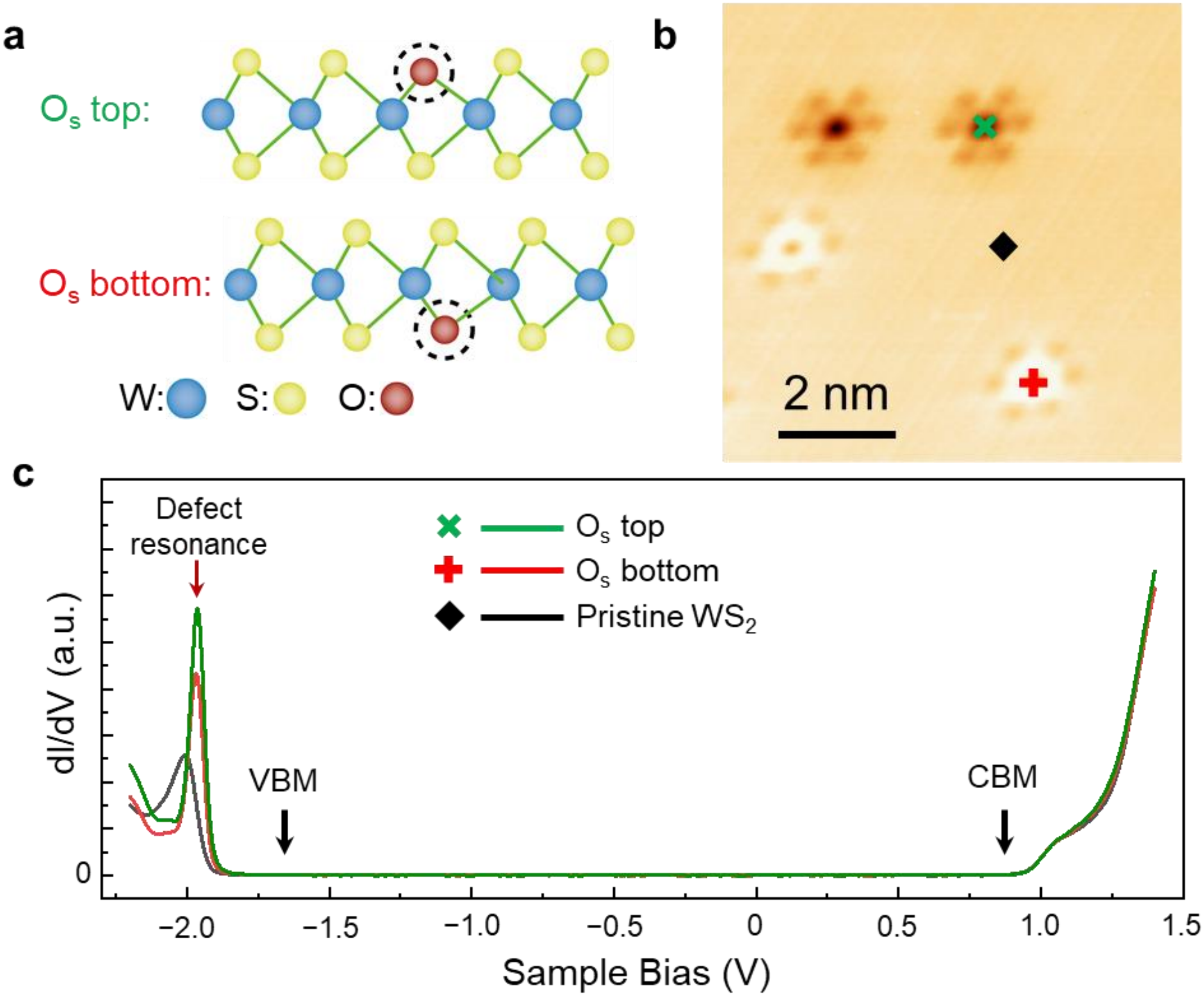

Supplementary Figure 8. STM/STS for O_S defect. (a) A schematic side view of oxygen substituting sulfur (O_S) in the top and bottom sulfur plane. (b) A typical STM image (V=1.3V, I=30pA) of as-grown monolayer WS_2 with O_S top and O_S bottom defects. (c) dI/dV spectrums recorded on O_S top (green), O_S bottom (red) defect, and pristine site (black) as marked in (b). The O_S defects show an equivalent bandgap to pristine WS_2 and a defect resonance deep inside the valence band, lacking of deep in-gap states.

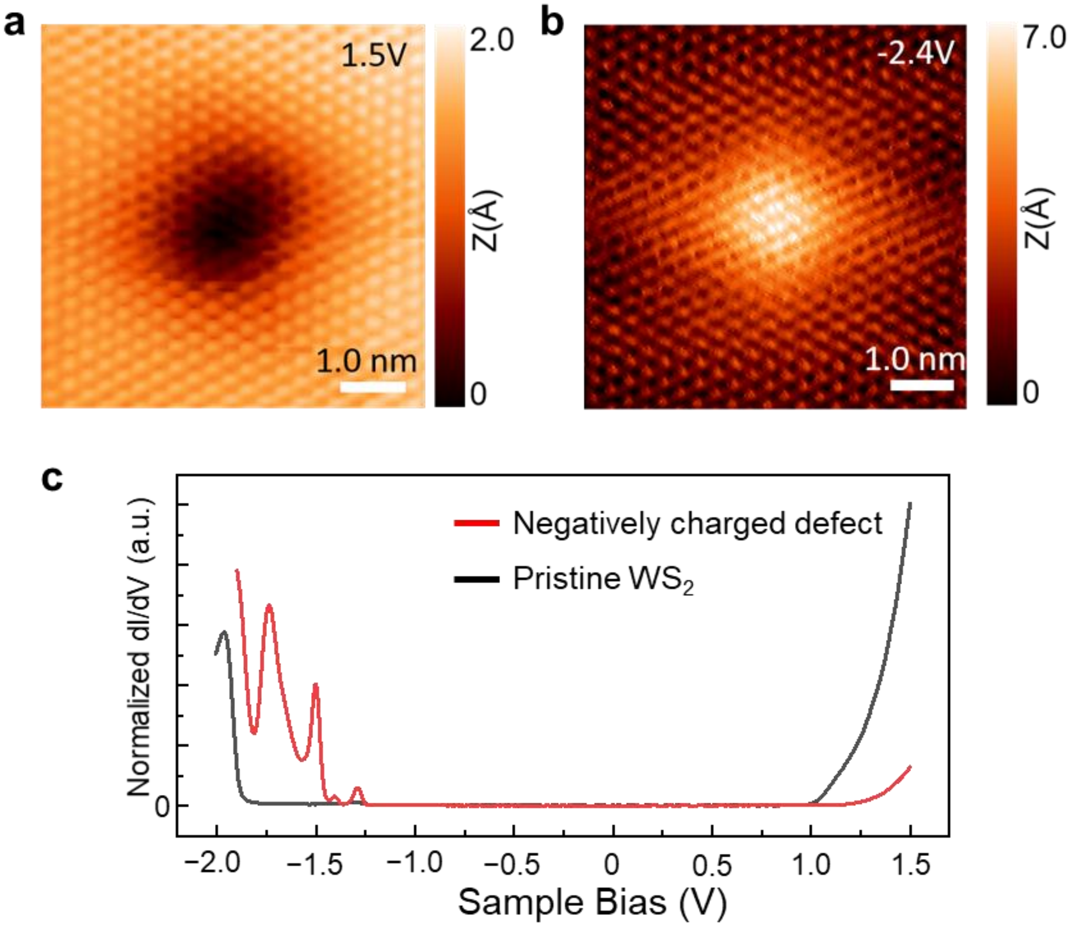

Supplementary Figure 9. STM/STS for negatively charged defect (NCD). (a) (b) Bias-dependent STM images of the NCD, appearing as a large depression at positive sample bias and a large protrusion at negative bias. (c) dI/dV spectrums on an NCD (red) and the pristine WS_2 (black), showing additional electronic resonances at negative sample bias.

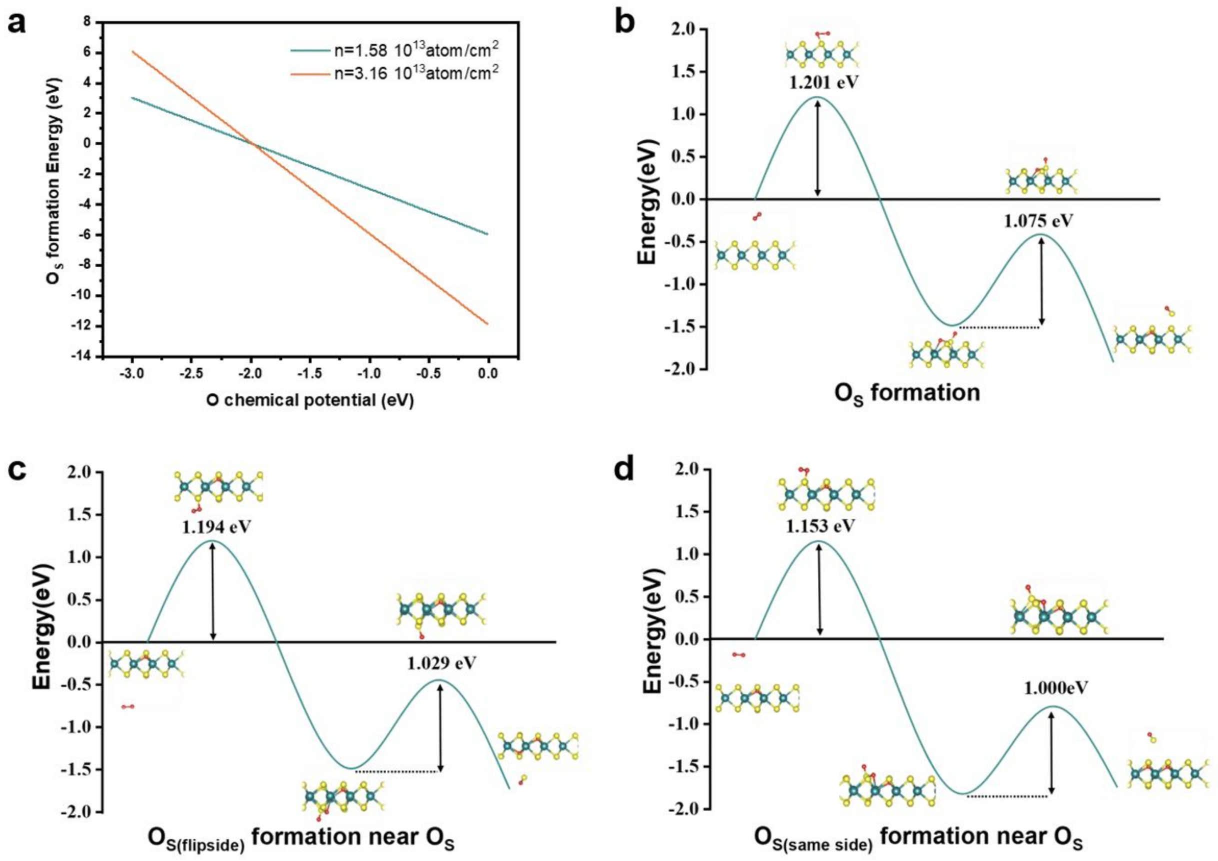

Supplementary Figure 10. Simulation of O_S defect formation. (a) Formation energy of O substituted by S (O_S) versus the chemical potential of O for two different O_S density. Reaction pathway and kinetic energy barriers of the O_2 dissociation and forming one O_S defect on the (b) pristine WS_2 crystal, (c) flipside near O_S and (d) same side near O_S.

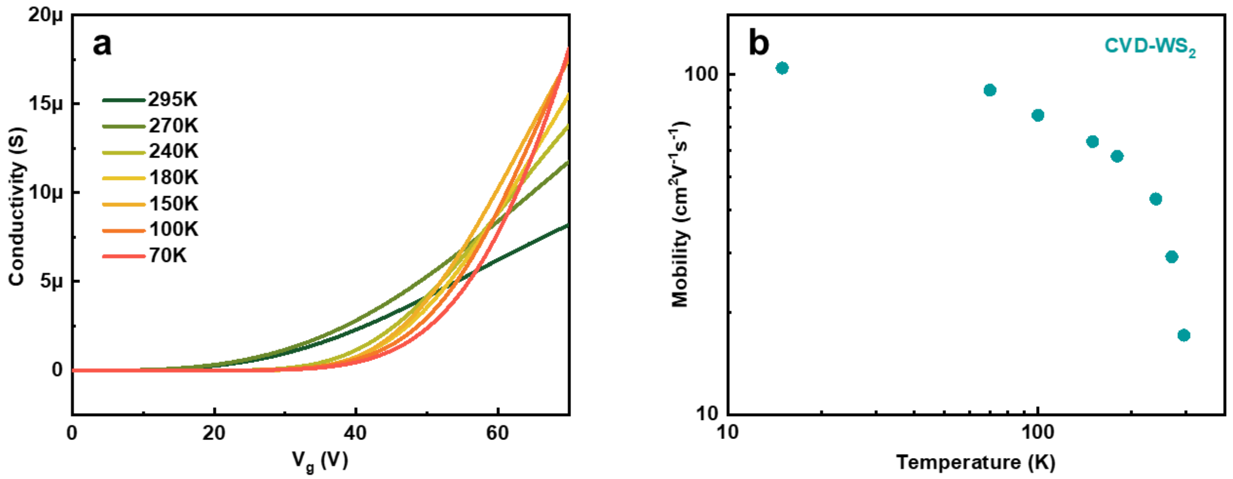

Supplementary Figure 11. Electrical performance of CVD-WS_2 monolayers. (a) Four-probe conductivity as a function of Vg for two cases of CVD-WS_2 monolayer device on the 300nm SiO_X substrate at different temperatures. (b) Electron field effect mobility of the corresponding CVD-WS_2 samples.

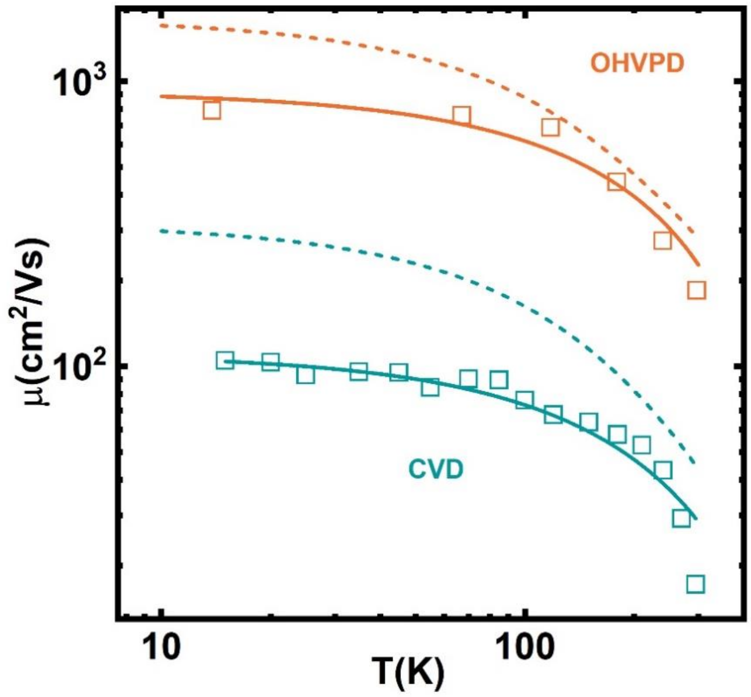

Supplementary Figure 12. Theoretical fitting of μ-T characteristic for OHVPD- and CVD-WS_2 devices under n= 4.3×10^{12} cm^{-2}. The squares are the experimental results. Solid lines are the best theoretical fitting as describe in the Method. The dashed lines are the calculated CI-dominated mobility.

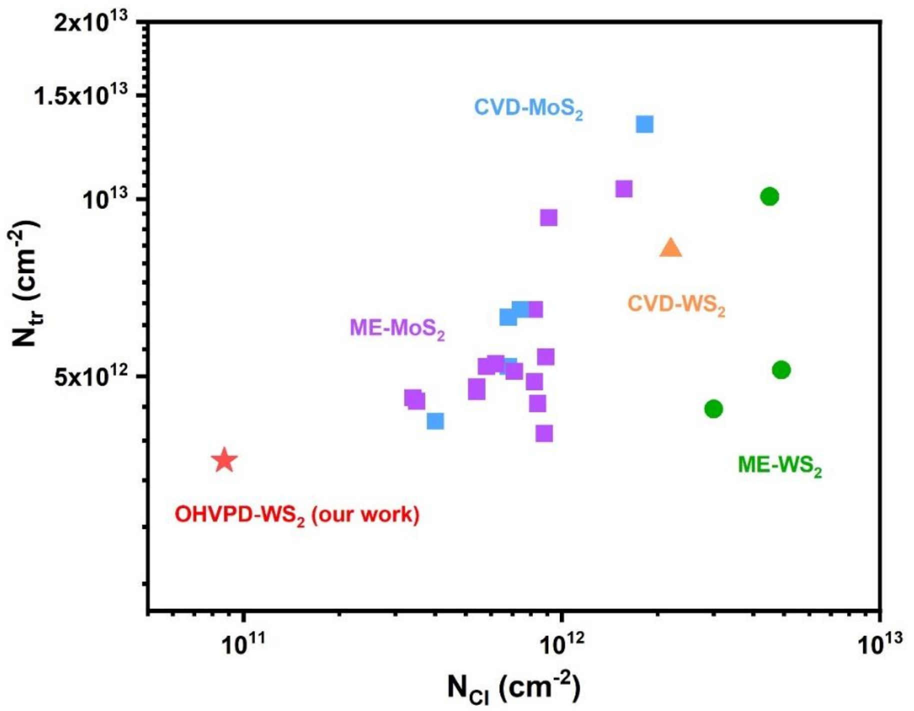

Supplementary Figure 13. Summary of reported N_{tr} and N_{CI} for MoS_2 and WS_2 monolayers with different fabrication technologies. The red start represents for our OHVPD-WS_2 shows the lowest N_{CI} as well as the lowest N_{tr} due to its relatively low defect density. (References are listed in Supplementary Table 1)

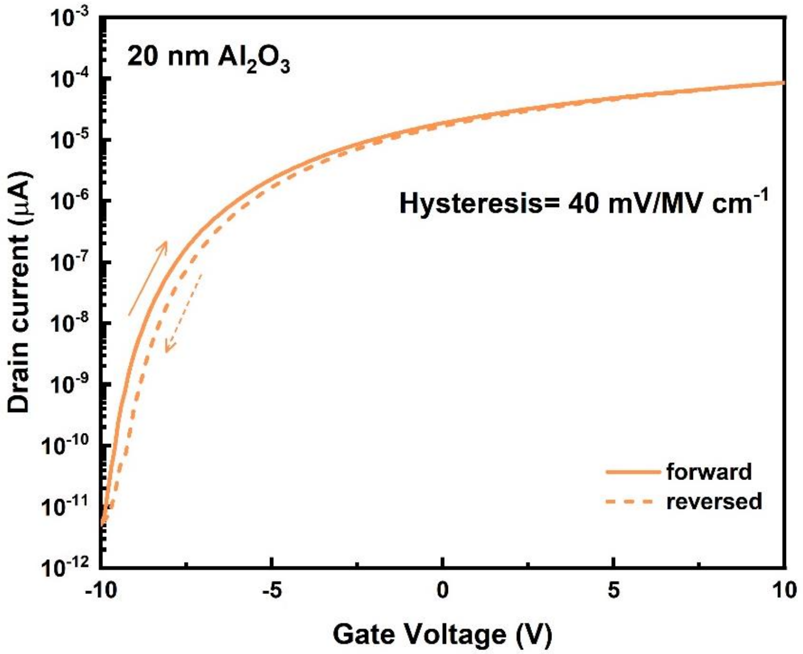

Supplementary Figure 14. Typical dual-sweep transfer curve characteristics of OHVPD-WS_2 monolayer device.

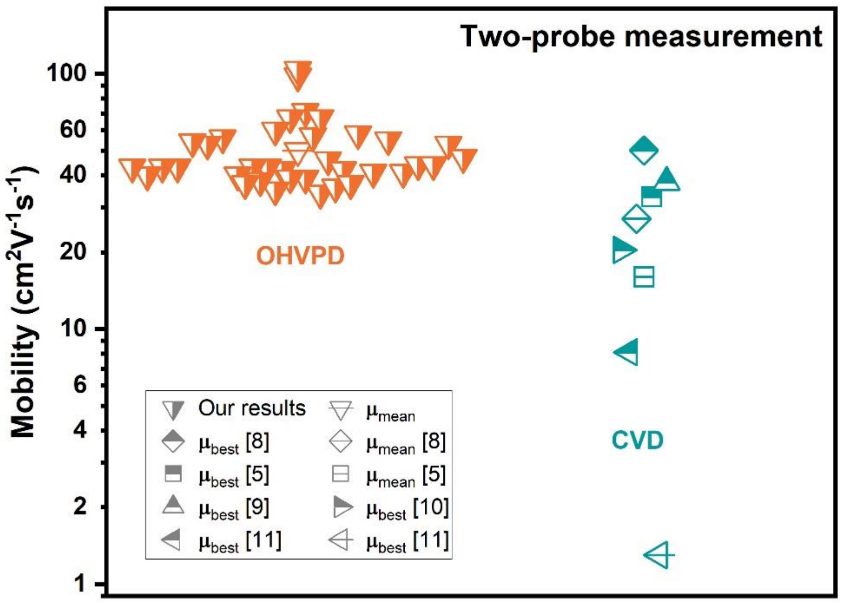

Supplementary Figure 15. Benchmarking field-effect mobility for WS_2 monolayers based on two-probe measurements.[5,8-11] The points with centerline interior represent the mean value.

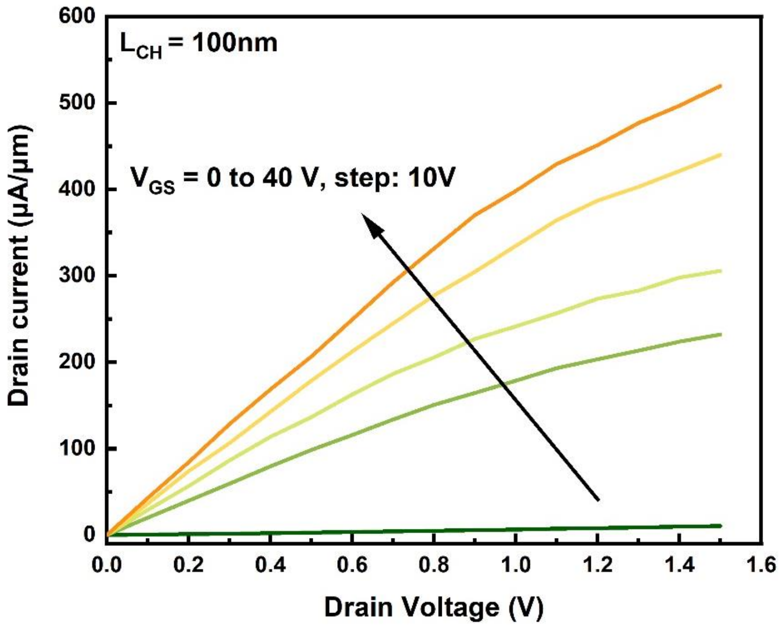

Supplementary Figure 16. The output characteristics of the short-gate-length (100 nm) FET based on OHVPD-WS_2.

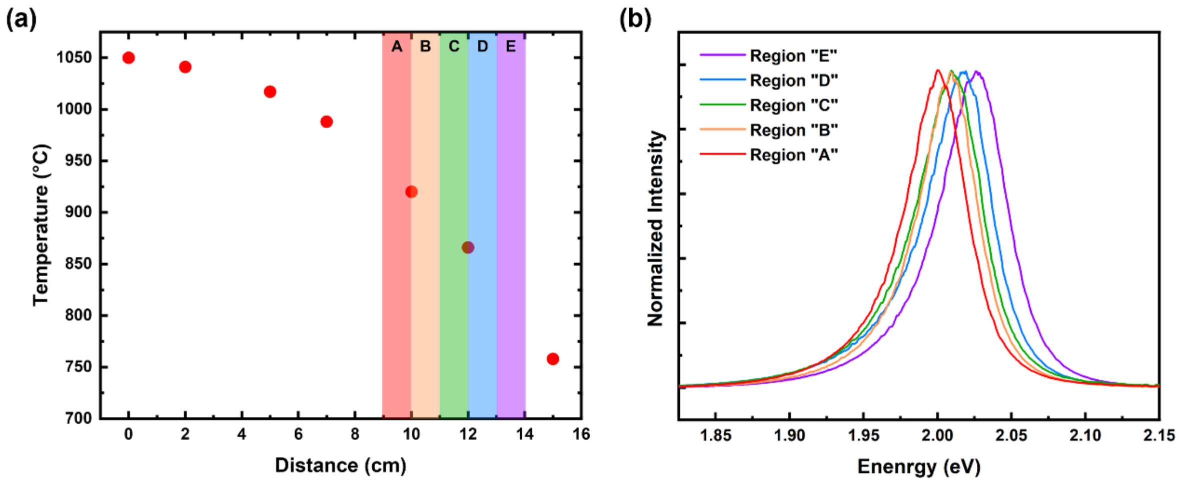

Supplementary Figure 17. Temperature and PL distributions. (a) Temperature profile of our single-heating zone furnace. (Setting temperature is 1050℃) (b) PL spectrums of OHVPD-WS_2 film from different regions.

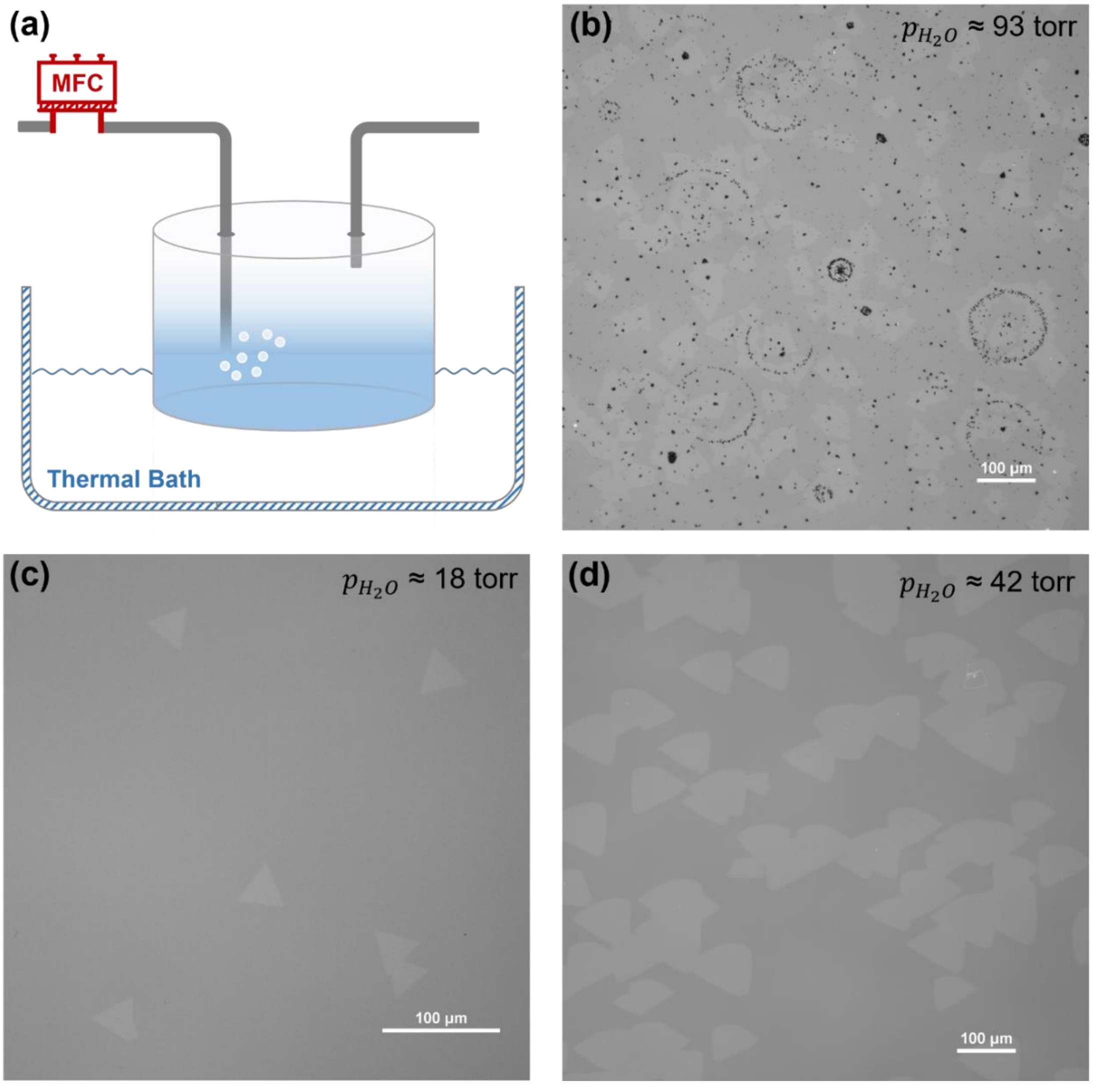

Supplementary Figure 18. Water vapor supply influence. (a) Schematic illustration of our bubbler set up for water vapor supply. Optical images of OHVPD-WS_2 growth results under (b) high p_{H_2O} (92.59 torr, 50℃), (c) low p_{H_2O} (17.54 torr, 20℃), and (d) optimized p_{H_2O} (42.20 torr, 35℃).

III. Supplementary Tables

Supplementary Table 1. Benchmarking FET performance of monolayer TMDCs.

Channel	Growth method	Electrode	Dielectric	Room temperature mobility [$cm^2V^{-1}s^{-1}$]	N_{tr} (10^{12} cm^{-2})	N_{CI} (10^{12} cm^{-2})	Ref.
1L-MoS_2	MPS-ME-MoS_2	Ti/Pd	Al_2O_3	113	4.5	0.84	[12]
1L-MoS_2	MPS-ME-MoS_2	Ti/Pd	Al_2O_3	101	4.0	0.88	[12]
1L-MoS_2	MPS-ME-MoS_2	Ti/Pd	HfO_2	125	5.4	0.89	[12]
1L-MoS_2	MPS-ME-MoS_2	Ti/Pd	HfO_2	149	4.9	0.82	[12]
1L-MoS_2	MPS-ME-MoS_2	Ti/Pd	SiO_2	34	6.5	0.82	[13]
1L-MoS_2	MPS-ME-MoS_2	Ti/Pd	SiO_2	81	5.1	0.71	[13]
1L-MoS_2	ME-MoS_2	MLG	t-BN/SiO_2	62	4.8	0.54	[14]
1L-MoS_2	ME-MoS_2	MLG	t-BN/SiO_2	62	4.72	0.54	[14]
1L-MoS_2	ME-MoS_2	MLG	d-BN/SiO_2	96	4.6	0.34	[14]
1L-MoS_2	ME-MoS_2	MLG	d-BN/SiO_2	84	4.54	0.35	[14]
1L-MoS_2	ME-MoS_2	MLG	SiO_2	72	5.2	0.58	[14]
1L-MoS_2	ME-MoS_2	MLG	SiO_2	55	5.25	0.62	[14]
1L-MoS_2	ME-MoS_2	Ti/Pd	SiO_2	23	9.3	0.91	[13]
1L-MoS_2	ME-MoS_2	Cr/Au	t-HfO_2	66	10.4	1.57	[15]

		-	t-HfO_2	48	-	2.5	16
		-	SiO_2	20	-	1.5	16
	MOCVD-MoS_2	Ti/Au	SiO_2	30	5.2	0.68	17
	CVD-MoS_2	MLG	d-BN/SiO_2	65	4.2	0.4	18
		Au	SiO_2	65.3	6.5	0.74	19
				66.4	6.3	0.68	20
				4.03	-	22.3	21
			t-Al_2O_3	24	13.4	1.82	22
	SAMs-CVD-MoS_2	Au	SiO_2	42.2	-	3.6	21
1L-WS_2	ME-WS_2	Ti/Pd	SiO_2	25	10.1	4.5	23
			Al_2O_3	49	5.13	4.9	23
				83	4.4	3.0	23
		-	SiO_2	40	-	1.35	16
	CVD-WS_2	Al/Au	SiO_2	17	8.2	2.2	our work
	OHVPD-WS_2	Al/Au	SiO_2	198	3.6	0.087	our work

IV. Supplementary References

1 Kilpatrick, M. & Lott, S. K. Reaction of flowing steam with refractory metals: III. Tungsten (1000–1700 C). *Journal of the Electrochemical Society* **113**, 17 (1966).

2 Sabourin, J. L. & Yetter, R. A. High-Temperature Oxidation Kinetics of Tungsten-Water Reaction with Hydrogen Inhibition. *Journal of Propulsion and Power* **27**, 1088-1096, doi:10.2514/1.B34093 (2011).

3 CHANG, L. L. Y. & PHILLIPS, B. Phase Relations in Refractory Metal-Oxygen Systems. *Journal of the American Ceramic Society* **52**, 527-533, doi:https://doi.org/10.1111/j.1151-2916.1969.tb09158.x (1969).

4 Berkdemir, A. *et al.* Identification of individual and few layers of WS2 using Raman Spectroscopy. *Scientific Reports* **3**, 1755, doi:10.1038/srep01755 (2013).

5 Sebastian, A., Pendurthi, R., Choudhury, T. H., Redwing, J. M. & Das, S. Benchmarking monolayer MoS2 and WS2 field-effect transistors. *Nature Communications* **12**, 693, doi:10.1038/s41467-020-20732-w (2021).

6 Chang, C.-H., Fan, X., Lin, S.-H. & Kuo, J.-L. Orbital analysis of electronic structure and phonon dispersion in MoS2, MoSe2, WS2, and WSe2 monolayers under strain. *Physical Review B* **88**, 195420, doi:10.1103/PhysRevB.88.195420 (2013).

7 Lide, D. R. *CRC handbook of chemistry and physics*. Vol. 85 (CRC press, 2004).

8 Aji, A. S., Solís ‐ Fernández, P., Ji, H. G., Fukuda, K. & Ago, H. High mobility WS2 transistors realized by multilayer graphene electrodes and application to high responsivity flexible photodetectors. *Advanced Functional Materials* **27**, 1703448 (2017).

9 Yue, Y. *et al.* Two-Dimensional High-Quality Monolayered Triangular WS2 Flakes for Field-Effect Transistors. *ACS Applied Materials & Interfaces* **10**, 22435-22444, doi:10.1021/acsami.8b05885 (2018).

10 Yun, S. J. *et al.* Synthesis of Centimeter-Scale Monolayer Tungsten Disulfide Film on Gold Foils. *ACS Nano* **9**, 5510-5519, doi:10.1021/acsnano.5b01529 (2015).

11 Chen, J. *et al.* Synthesis of Wafer-Scale Monolayer WS2 Crystals toward the Application in Integrated Electronic Devices. *ACS Applied Materials & Interfaces* **11**, 19381-19387, doi:10.1021/acsami.9b04791 (2019).

12 Yu, Z. *et al.* Analyzing the Carrier Mobility in Transition-Metal Dichalcogenide MoS2 Field-Effect Transistors. *Advanced Functional Materials* **27**, 1604093, doi:https://doi.org/10.1002/adfm.201604093 (2017).

13 Yu, Z. *et al.* Towards intrinsic charge transport in monolayer molybdenum disulfide by defect and interface engineering. *Nature Communications* **5**, 5290, doi:10.1038/ncomms6290 (2014).

14 Liu, Y. *et al.* Toward Barrier Free Contact to Molybdenum Disulfide Using Graphene Electrodes. *Nano Letters* **15**, 3030-3034, doi:10.1021/nl504957p (2015).

15 Radisavljevic, B. & Kis, A. Mobility engineering and a metal–insulator transition in monolayer MoS2. *Nature Materials* **12**, 815-820, doi:10.1038/nmat3687 (2013).

16 Lee, Y., Fiore, S. & Luisier, M. in *2019 IEEE International Electron Devices Meeting (IEDM).* 24.24.21-24.24.24.

17 Kang, K. *et al.* High-mobility three-atom-thick semiconducting films with wafer-scale homogeneity. *Nature* **520**, 656 (2015).

18 Cui, X. *et al.* Multi-terminal transport measurements of MoS2 using a van der Waals heterostructure device platform. *Nature Nanotechnology* **10**, 534-540, doi:10.1038/nnano.2015.70 (2015).

19 Jariwala, D. *et al.* Band-like transport in high mobility unencapsulated single-layer MoS2 transistors. *Applied Physics Letters* **102**, 173107, doi:10.1063/1.4803920 (2013).

20 Sangwan, V. K. *et al.* Low-Frequency Electronic Noise in Single-Layer MoS2 Transistors. *Nano Letters* **13**, 4351-4355, doi:10.1021/nl402150r (2013).
21 Wan, X. *et al.* Quantitative Analysis of Scattering Mechanisms in Highly Crystalline CVD MoS2 through a Self-Limited Growth Strategy by Interface Engineering. *Small* **12**, 438-445, doi:https://doi.org/10.1002/smll.201502392 (2016).
22 Sanne, A. *et al.* Top-gated chemical vapor deposited MoS2 field-effect transistors on Si3N4 substrates. *Applied Physics Letters* **106**, 062101, doi:10.1063/1.4907885 (2015).
23 Cui, Y. *et al.* High-Performance Monolayer WS2 Field-Effect Transistors on High-κ Dielectrics. *Advanced Materials* **27**, 5230-5234, doi:https://doi.org/10.1002/adma.201502222 (2015).
